# Acid–Base Dysregulation Links Aging Metabolism to Frailty

**DOI:** 10.1111/acel.70466

**Published:** 2026-04-14

**Authors:** Wan‐Hui Liao, Wolfgang Langhans, Maciej Henneberg

**Affiliations:** ^1^ Division of Geriatric Medicine, Department of Internal Medicine MacKay Memorial Hospital Taipei Taiwan; ^2^ Physiology and Behavior Laboratory ETH Zurich Schwerzenbach Switzerland; ^3^ Institute of Evolutionary Medicine, Medical Faculty University of Zurich Zurich Switzerland; ^4^ Biological Anthropology and Comparative Anatomy Unit Adelaide University Adelaide Australia

## Abstract

Frailty denotes a state of high vulnerability and, as proposed by Fried and colleagues, arises from “energetic collapse” across multiple physiological systems, in which altered energy metabolism undermines resilience. We suggest that dysregulation of acid–base balance represents a critical yet underappreciated mechanism driving this collapse. With aging, cumulative stress burden diminishes the capacity for intracellular and extracellular acid buffering, renal acid excretion and ventilatory reserve, leading to impaired pH homeostasis, reduced mitochondrial ATP production, and declining cellular and organismal efficiency. Skeletal muscle, bone, liver, and kidney cooperate to mobilize the base reserves and redirect amino acid metabolism to enhance renal acid elimination. But this adaptation occurs at the expense of musculoskeletal integrity—a hallmark of aging. The shrinking iceberg metaphor illustrates frailty progression. Repeated stressors erode the acid buffer and energy reserve. Incorporating acid–base dysregulation into frailty models highlights new therapeutic targets—including diet, exercise and buffering strategies to preserve reserve and delay frailty progression.

## Graphical abstract

Our Metabolic Buffer Model posits that chronic acid load from aging and accumulated stressors depletes total acid buffer reserve, disrupting intersystem crosstalk. This hidden homeostatic erosion drives clinical frailty and underlies homeostatic failure during stress, unifying Rockwood's deficit accumulation and Fried's phenotype models.

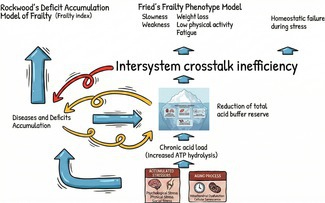

## 1 Introduction

Frailty in old age is hallmarked by a heightened vulnerability to stressors due to a reduced physiological reserve that, in younger years, allows the body to withstand and recover from stressors while preserving homeostasis. Frailty predicts high risks of falls, hospitalization, disability, poor prognosis, including delayed recovery from illness, and death (Ensrud et al. 2009; Xue 2011). There have been two prevailing definitions of frailty so far: the phenotypic frailty model and the deficit accumulation model. Linda Fried and colleagues in 2001 proposed a phenotypically based definition that comprises clinical traits of slow gait speed, weak grip strength, low physical activity, fatigue, and unintentional body weight loss (Fried et al. 2001). The presence of three or more of those five traits is categorized as “frail”, one to two items as “prefrail”, and zero items as “robust”.

In contrast, Kenneth Rockwood and colleagues assessed frailty as an accumulation of deficits across several health domains and presented it as a frailty index. It takes into account comorbidities, cognitive and physical disabilities, functional limitations, sensory impairments (e.g., vision or hearing loss) and abnormal laboratory findings (Mitnitski et al. 2001). These deficits commonly increase with age and are associated with adverse outcomes. Each deficit is weighted equally, and a higher frailty index indicates a greater frailty. The frailty index model has been shown to be more sensitive than the phenotypic model in predicting adverse health outcomes such as mortality and institutionalization (Rockwood et al. 2007). However, this index is a composite of a number of deleterious changes occurring in various body systems due to various causes occurring with advancing age, serving primarily as a descriptive tool. Thus, it does not address the common underlying pathophysiology of frailty and therefore offers limited guidance for interventions to improve this status. Nonetheless, such an index is useful to describe the overall clinical status of a patient. A greater frailty index essentially illustrates a greater lifetime burden and the accumulation of stressors.

Fried, on the other hand, viewed frailty as fundamentally an energetic problem (Fried et al. 2021). It is a progressive, energy‐inefficiency driven collapse of integrity across multiple interrelated physiological systems, primarily involving the musculoskeletal, metabolic, appetite control, and stress‐response systems (including the hypothalamus‐pituitary–adrenal (HPA) axis and the autonomic nervous system). In the normal physiological state, these systems work coordinately to maintain homeostasis by (1) anticipating and counteracting environmental stressors before they cause major homeostatic disturbances or by (2) responding to stressors efficiently to minimize the disturbances caused. Overall, these mechanisms demonstrate robustness, a capacity to endure and to recover from stress, and adaptability, that is, the capacity to adjust, reorganize, or modify function in response to stress.

Aging and accumulated stress disrupt the efficiency of communication and coordination between cells and systems, gradually undermining physiological integrity that eventually crosses a severity threshold, precipitating a state of diminished function and resilience, that is, physical frailty (Fried et al. 2021). A spectrum of characteristic derangements emerges during the progression of frailty, including impaired mitochondrial oxidation, cellular senescence, poor intercellular communication (Lopez‐Otin et al. 2023), chronic inflammation (Leng et al. 2007; Soysal et al. 2016), and delayed phosphocreatine recovery time (Varadhan et al. 2019). Additionally, there are exaggerated responses to glucose (Kalyani et al. 2012) and corticotropin challenge (Le et al. 2021), vaccine failure (Yao et al. 2011), and orthostatic hypotension (O'Connell et al. 2015). These physiological derangements are reflected clinically as fatigue, sarcopenia, repeated infection, and diabetes, which are commonly observed in frail individuals. Fried observes that inefficient communication and cooperation among systems compromise dynamic functioning in frailty, linking aging and accumulated stress to the emergence of frailty in a hierarchical and non‐linear manner (Fried et al. 2021).

Fried's frailty model of dysregulated complex dynamical systems also emphasizes that frailty is not simply a state of energy deficit but rather a consequence of impaired energy production, distribution, and regulation. This is consequential to chronic low‐grade inflammation that impairs mitochondrial oxidation, inducing insulin resistance and altering HPA axis activity. Consistent with this view, simple provision of excess calories or nutrients often fails to restore robustness (Gray‐Donald et al. 1995)—indeed, overfeeding can exacerbate frailty by increasing metabolic burden, promoting inflammation, or contributing to sarcopenic obesity (Klein et al. 1998).

## 2 Reduced Acid Buffering May Account for Reduced Physiological Reserve in Frailty

Energetic efficiency is central to staying robust and to survival and reproductive success (Liao et al. 2016). For homeothermic humans, it is built on an optimal internal temperature and optimal pH, both of which are critical for maximizing enzymatic activity. Enzyme function underlies most biochemical reactions, including mitochondrial ATP production (Toth et al. 2020), the maintenance of membrane stability (Kandrashina et al. 2024), membrane potential, and physiological performance. Overall, pH homeostasis at the intracellular and extracellular levels is preserved through complementary buffer systems and by regulation of cellular metabolism and activity, which are subject to both endocrine and behavioral control. Buffer systems such as the bicarbonate system provide the first line of defense against rapid pH fluctuations. Lungs respond within minutes by changes in ventilation, while the kidneys provide long‐term regulation through excreting protons (H^+^) and generating new bicarbonate (Liao et al. 2018).

Accumulating epidemiologic evidence indicates that even “mild” deviations in serum bicarbonates have clinically meaningful implications for aging people. Observational studies have linked serum bicarbonate below 25 mEq/L—a threshold often considered clinically normal—to impaired physical performance, including slower gait speed, reduced muscle strength, and altered gait mechanics (Abramowitz et al. 2011; Ho et al. 2022). Longitudinal data from *The Health*, *Aging*, *and Body Composition Study* of initially well‐functioning older adults (ages 70–79) further established low serum bicarbonate as an independent predictor of incident, persistent lower‐extremity functional limitation (Yenchek et al. 2014). Notably, low bicarbonate level remains a significant risk factor for mortality even in individuals with preserved glomerular filtration rate (GFR > 60 mL/min/1.73 m^2^). The associations persist even with bicarbonate levels in the low‐normal clinical range.

While lower bicarbonate levels are correlated with declining GFR, they also occur frequently in older adults with preserved renal function (Yenchek et al. 2014). In these cases, low bicarbonate plausibly reflects a mild or subclinical metabolic acidosis. The mechanistic association between acidosis and physical decline is thought to involve derangements in skeletal muscle metabolism that promote sarcopenia‐ the progressive loss of skeletal muscle mass, quality, and strength. These acidosis‐induced derangements include catabolic signaling (Pang et al. 2023; Su et al. 2017), insulin resistance (Cree et al. 2004; Garibotto et al. 2015), increased inflammatory cytokines (Addison et al. 2014; Verzola et al. 2017), mitochondrial dysfunction and oxidative stress (Andreux et al. 2018; Gamboa et al. 2016; Peterson et al. 2012; Wawrzyniak et al. 2016), features that are also reported in the aging‐related frailty phenotype. In comparison to aging, this catabolic process occurs more rapidly in chronic kidney disease (CKD) because of not only the greater severity of acidosis but also the accumulation of uremic toxins, such as indoxyl sulfate and p‐cresyl sulfate (K. Wang et al. 2023). Based on in vitro evidence, these toxins inhibit myogenic differentiation (Alcalde‐Estevez et al. 2021) and damage mitochondria (Enoki et al. 2017), further accelerating sarcopenia in CKD. On this basis, Wesson et al. (2022) proposed that acidosis‐induced metabolic derangement in skeletal muscle also represents a key driver in aging‐related frailty. However, it is critical to distinguish established mechanism from clinical hypothesis: while the cellular pathways are well‐characterized in animal and CKD models, we lack longitudinal human data. Without tracking pH against frailty onset, it remains unclear if acidosis is a driver of frailty, a marker of its presence, or a consequence of muscle wasting (via loss of intracellular buffers).

We hypothesize that the reduced total acid buffer reserve is the fundamental physiological underpinning of frailty. Given that bicarbonate is only one factor of pH homeostasis, its subclinical depletion might represent a broader erosion of the physiological resilience. Physical and psychological stressors drive highly energy‐consuming biochemical processes, increasing ATP hydrolysis and the subsequent production of H^+^ (Robergs et al. 2004). We posit that this increased metabolic acid load unmasks critical vulnerability in the frail individual: a reduction in “total” acid buffering capacity (both bicarbonate and non‐bicarbonate buffers) to below a critical threshold. This systemic inability to neutralize acute acid stress precipitates homeostatic failure. Maintaining pH within a narrow homeostatic range is a prerequisite for physiological robustness, ensuring that immune, endocrine, hemostatic, and cognitive systems can interact efficiently during a stress response. Conversely, a depleted buffering capacity compromises this adaptability; when the body cannot buffer the proton load of a stressor, the resultant acidosis disrupts this inter‐system crosstalk, leading to system‐wide collapses. We therefore propose expanding Fried's model to include acid–base dysregulation. This proposed “metabolic buffer model” serves as a missing link, connecting the cumulative burden of aging and chronic stress (the accumulation of deficits) to the precipitous systemic energetic collapse (the phenotype) observed in frailty (graphical abstract).

## 3 Aging‐Related Mitochondrial Respiration Insufficiency Contributes to Excess Intracellular Acid Production, Inflammation and Cell Damage

Mitochondrial dysfunction—a hallmark of aging—impairs respiratory chain complexes, reducing their capacity for electron transfer from NADH and FADH_2_ and thereby compromising the generation and maintenance of the proton gradient across the inner mitochondrial membrane. This disruption disables efficient ATP synthesis via oxidative phosphorylation and leads to accumulation of ADP and AMP, which activate glycolysis. Simultaneously, the inability of the electron transport chain to oxidize NADH and FADH_2_ results in increased NADH/NAD^+^ and FADH_2_/FAD^+^ ratios. NADH accumulation in the cells has recently gained huge research interest because it is not merely a metabolite, but it can form NADH reductive stress, which reprograms cellular metabolism (Yang et al. 2025). The excess NADH inhibits pyruvate dehydrogenase through activation of pyruvate dehydrogenase kinase, further blocking pyruvate entry to tricarboxylic acid cycle (TCA) (Pettit et al. 1975). The overall effects are that cells shift toward increased glycolysis to obtain energy with a greater reliance on nonmitochondrial pathways, such as anaerobic glycolysis and the phosphagen system (also known as the ATP‐PCr or creatine phosphate system) (Robergs et al. 2004). The phosphagen system is particularly important in skeletal and cardiac muscles, where it provides immediate energy. During high intensity work, once the intracellularly stored ATP (3–8 mmol/kg muscle) is depleted, cells rely on phosphocreatine (PCr) (10–40 mmol/kg muscle) for very short bursts of activity (seconds), then shift to glycogen breakdown and anaerobic glycolysis for slightly longer and high‐intensity activity. However, these systems are limited by low intramuscular reserves. The muscle cells also take and metabolize circulating glucose to sustain ATP production. Unlike mitochondrial respiration which creates and utilizes the proton gradient across the inner mitochondrial membrane to drive efficient ATP production, both glycogenolysis and glycolysis in the cytosol are not only energetically less efficient, but also lead to excess proton generation and intracellular acidosis (Robergs et al. 2004).

Intracellular acidosis disrupts cellular homeostasis and accelerates several hallmarks of aging, including genomic instability, telomere attrition, epigenetic alteration, loss of proteostasis, and impaired macroautophagy (Antosiewicz and Kane 2022). Furthermore, dysfunctional respiratory chain complexes promote proton and electron leakage, increasing reactive oxygen species formation and risking mitochondrial membrane permeabilization. Therefore, intracellular acidosis and reactive oxygen species amplify mitochondrial dysfunction and may initiate inflammation and cell death (Amorim et al. 2022).

To neutralize the harmful effects of intracellular acid accumulation, cells rely on intracellular acid buffers, whose composition and capacity vary by cell type, and are generally proportional to metabolic activity and acid load. Skeletal muscle and blood cells have higher intracellular acid buffer capacity. Histidine‐rich proteins are the predominant intracellular acid buffers across the cell type, followed by phosphate (H_2_PO_4_^−^/HPO_4_^2−^) (Shaw and Gregory 2022). Skeletal muscle cells additionally use carnosine (del Favero et al. 2012; Matthews et al. 2019) while cardiac muscle cells use bicarbonate to augment acid buffer capacity (Vaughan‐Jones et al. 2009; Wang et al. 2014). Red blood cells rely primarily on hemoglobin with a secondary contribution from bicarbonate (Salenius 1957). Excess intracellular acid (H^+^) is exported via membrane transporters (e.g., V‐type H^+^ ATPases, Na^+^/H^+^ exchangers, Na^+^‐HCO_3_^−^ cotransporters) or converted to CO_2_ for diffusion (Seifter and Chang 2017). Plasma bicarbonate, proteins, in particular albumin, and phosphate together with red blood cells constitute the main extracellular acid buffer system (Salenius 1957). This complex system facilitates the transport of excess acid to kidneys and lungs for eventual elimination (Seifter and Chang 2017).

## 4 Declining Acid Buffering Capacity and Increasing Metabolic Cost With Age

### 4.1 Intracellular and Extracellular Decline

Histidine‐rich proteins and carnosine serve as the primary intracellular buffers in skeletal muscle. Changes in the levels of muscular carnosine influence exercise performance (Baguet et al. 2010). Notably, muscular carnosine levels have been shown to decline with aging (Stuerenburg and Kunze 1999), contributing to limited intracellular buffering capacity and therefore compromising physical performance and muscular function. Carnosine supplementation in elderly improves physical capacity (del Favero et al. 2012). Similarly, extracellular buffering capacity diminishes with age. Aging is also associated with a progressive reduction in serum albumin by roughly 0.01–0.015 g/dL per year after age 65 (Gom et al. 2007) as well as reductions in hemoglobin levels (Salive et al. 1992), even in otherwise healthy individuals. Both serum albumin and hemoglobin are also independently correlated with physical function (Aung et al. 2011). Since these proteins are key components of the “non‐bicarbonate” buffer system, their concurrent decline with aging erodes the body's resilience to acid stress.

### 4.2 Compromised Systemic Elimination

Systemically, aging is associated with a gradual decline in respiratory and renal functions, compromising the body's capacity to eliminate acid efficiently (Lalley 2013; Papacocea et al. 2021). In fact, according to an observational study, the prevalence of subclinical metabolic acidosis, defined as bicarbonate < 23 mEq/L in adults over 50, reaches 22% (Abramowitz et al. 2011). It is suggested to be often driven by diet or defects in renal acidification‐ in particular, type IV renal tubular acidosis, or age‐related tubular inefficiency (Frassetto et al. 1996; Quaglia et al. 2014)—rather than glomerular failure.

### 4.3 The Rising Metabolic Cost of Stress

In parallel, cumulative psychosocial and physiological stressors—including multimorbidity, functional and cognitive decline, bereavement and major life transitions, caregiving burdens, and social or financial adversity contribute to chronic activation and remodeling of both the sympatho‐adrenomedullary system (SAM) and the HPA axis—producing habituation to repeated stimuli but sensitization to novel stressors, adrenal hypertrophy, corticotropin hypersensitivity, and excess glucocorticoid output. This may explain why frail elderly adults often display elevated basal glucocorticoid levels, flattened cortisol rhythm (Varadhan et al. 2008), increased sympathetic tone (Rowe and Troen 1980), and heterogeneous stress reactivity—blunted to repeated/predictable stressors but exaggerated to novel or unpredictable challenges (McEwen 1998). In addition to hormonal imbalance, chronic stress contributes to frailty also through the induction of chronic inflammation, oxidative stress, mitochondrial dysfunction, DNA damage and cell senescence (El Assar et al. 2020). Chronic inflammation drives sustained immune cell activation and turnover, continuous cytokine synthesis and secretion, and activation of hepatic acute‐phase response. Oxidative stress necessitates persistent activation of redox defense systems, including antioxidant regeneration, and the repair and replacement of oxidatively damaged molecules. DNA damage further elicits energy‐dependent DNA repair processes. In cellular senescence, cells are non‐dividing and do not contribute to tissue function but they remain metabolically active (Wiley and Campisi 2021).

Collectively, these responses represent a high energy‐demand state, raising ATP turnover and glycolytic flux, thus amplifying intracellular and systemic acid load (Hermann, Biallas, et al. 2019; Hermann, Lay, et al. 2019; Robergs 2021). Sensory impairments, though often overlooked as causes of neuroendocrine activation, heavily compromise energetic efficiency. Navigating the environment with compromised sensory input requires more vigilance and cognitive processing. The brain and skeletal muscle must also exert extra efforts to interpret ambiguous signals, maintain balance, and ensure safety. Therefore, the convergence of impaired intracellular and extracellular acid buffering, compromised renal and respiratory elimination of acid, and the metabolic cost of chronic stress ultimately overwhelms acid–base regulatory capacity in aging.

## 5 Physical Frailty Syndrome Is a Maladaptive Consequence of Acid Overload

Gradual acid accumulation likely drives adaptive reductions in physical activity and energy expenditure—manifesting clinically as fatigue and a slowed pace—strategies to limit further acid production. It can be viewed as a regulated effort to preserve intracellular and extracellular homeostasis and promote survival (McKenna and Hargreaves 2008). While muscle glycogen depletion initiates fatigue, acidosis within muscle tissue intensifies it and impairs contractile efficiency (Allen et al. 1995; Chycki et al. 2018). Prolonged muscular acidosis disrupts mitochondrial oxidative phosphorylation, reduces ATP production, promotes proteolysis and may even trigger myocyte death (Ho and Abramowitz 2022). Furthermore, acidosis interferes with thyroid hormone metabolism at the tissue level (Narasaki et al. 2021). By suppressing deiodinase and therefore T3 availability and reducing tissue responsiveness to TSH stimulation (Wiederkehr et al. 2004), acidosis can exacerbate fatigue and weakness. Recovery could also be compromised. Acidosis interferes with insulin‐stimulated glycogen synthase, hindering glycogen repletion (Baldini and Avnet 2018; Bouskila et al. 2010), and damages satellite cells, which are essential for muscle regeneration. Oxidative stress‐induced satellite cell damage impairs self‐renewal and differentiation into myogenic progenitors. These changes accelerate the development of sarcopenia and muscle weakness (Q. Yang and Chan 2022). In turn, muscle loss and sarcopenia deplete carnosine, phosphate and histidine‐rich proteins, diminishing intracellular acid‐buffering capacity and amplifying the effects of low‐grade metabolic acidosis on muscular dysfunction.

## 6 Clinical Vulnerability of Frailty Is due to Reduced Acid‐Buffering Systems

Just as chronic localized or low‐grade systemic acidosis impairs renal and respiratory function, it can compromise other organs and further destabilize systemic acid–base imbalance through inefficient metabolism—requiring more ATP than normal to sustain basic cellular work. In combination with the aforementioned NADH‐driven reductive stress (R. Yang et al. 2025), the inefficiency further burdens mitochondrial and metabolic reserve. The consequences can be cognitive dysfunction (Marmarou 1992), immune anergy (Kellum et al. 2004), impaired gastrointestinal processing and uptake of nutrients as well as altered intestinal motility (Grant et al. 2017; Holzer 2007).

To mitigate extracellular acid accumulation in the face of compromised normal acid–base buffer systems, the body mobilizes skeletal reserves as a major substituting buffering mechanism. Although bone cells contribute little to intracellular buffering, the mineralized extracellular matrix provides substantial capacity. Acute acid loading liberates alkaline compounds—particularly hydroxyapatite and carbonate apatite—into the circulation with unbuffered protons displacing basic ions in chemical reactions such as Ca_10_(PO_4_)_6_(OH)_2_ + 8H^+^ → 10Ca^2+^+ 6HPO_4_^2−^ + 2H_2_O; CaCO_3_ + H^+^ → Ca^2+^ + HCO_3_^−^ (Lemann Jr. et al. 2003). On the other hand, chronic low‐grade metabolic acidosis stimulates osteoclastic bone resorption and suppresses osteoblastic bone formation (Arnett 2003), driving progressive mineral loss and skeletal fragility (Bushinsky 2001).

Simultaneously, metabolic acidosis, even if only transiently induced by diet (Williams et al. 2016), could perturb endocrine signaling, not only promoting the aforementioned insulin resistance but also enhancing corticotropin‐dependent cortisol and aldosterone secretion (Perez et al. 1979) potentially via chemosensory activation of the HPA and SAM axis (Paleczny et al. 2014). In frailty, stress‐induced ATP hydrolysis can readily exceed the limited acid‐buffering reserves, precipitating acidosis, which activates chemosensory receptors in the carotid bodies and in the hypothalamic nuclei (Dempsey and Smith 2014). This leads to amplification of sympathetic outflow and cortisol secretion, thereby reinforcing a maladaptive stress cycle that generates additional protons. Although chemoreceptor activation also stimulates ventilation, if respiratory compensation cannot match the proton load from stress‐driven ATP turnover, systemic acidemia readily ensues (Liao et al. 2018; Milici‐Emili and Petit 1960).

Furthermore, acidosis can synergize with insulin resistance, hypercortisolism, and hyperaldosteronism to accelerate catabolism in bone and muscle (DiNicolantonio and O'Keefe 2021; May et al. 1986). Aldosterone levels are increased during acidosis (Wagner 2014). Consequently, sarcopenia, and osteoporosis frequently coexist with frailty, compounding risks of falls, fractures, disability, and mortality. At the same time, glucocorticoids and aldosterone also enhance renal acid excretion by stimulating ammoniagenesis through the induction of glutaminase, which converts glutamine to ammonia (NH_3_) and a‐ketoglutarate in the proximal tubule. The generated NH_3_ buffers H^+^ to form ammonium (NH_4_^+^), while α‐ketoglutarate metabolism contributes to bicarbonate regeneration. When dietary glutamine supply is insufficient, skeletal muscle proteolysis releases glutamine to sustain this process (King et al. 1983). Meanwhile, the liver downregulates ureagenesis, allowing a greater proportion of glutamine to be diverted from hepatic nitrogen disposal toward renal ammoniagenesis (Holecek 2024). In parallel, these hormones augment titratable acid excretion in the form of HPO4^2−^, and Na^+^/H^+^ exchange in the proximal tubule (Hamm et al. 1999). Collectively, these physiological adjustments may represent adaptive efforts among kidney‐liver‐muscle to stabilize systemic pH by mobilizing amino acid substrates and recruiting buffer reserves in bone. They do so, however, at the expense of structural integrity and resilience, accelerating the catabolic cycle characteristic of frailty.

In individuals with diminished acid‐buffering reserve, severe or prolonged stress—such as systemic infection or septic shock—can easily overwhelm systemic buffering mechanisms, resulting in metabolic acidosis characterized by elevated blood lactate and decreased pH. This systemic acidemia precipitates widespread physiological dysfunction and is closely linked to poor outcomes in frail individuals.

## 7 Lack of Natural Selection Expands Phenotypic Variation of Frailty

Frailty exhibits substantial phenotypic variation in both age of onset and severity. Beyond the inherited germline variants and the somatic genetic damage arising from repeated life stressors, the absence of natural selection in post‐reproductive life further broadens the variation. Though spontaneous mutations at any single locus in the human genome occur with relatively low frequencies (10^−4^–10^−8^), each neonate is estimated to carry approximately 50–90 de novo mutations (Jonsson et al. 2017), some of which could be mildly detrimental. Effects of these spontaneous mutations, together with mutations induced or unmasked by environmental or physiological stressors, accumulate progressively with advancing age.

Evolutionary adaptations that safeguard organismal homeostasis are shaped by natural selection, which operates through differential reproductive success and maintains a mutation‐selection balance. However, once reproduction ceases, natural selection no longer constrains late‐life physiology. The accumulated mutations across genomes render late‐life biological processes less adaptive, disrupting homeostasis of vital processes and increasing vulnerability to stress. Moreover, many genes have pleiotropic effects: alleles that enhance reproductive performance and thus undergo positive selection may simultaneously predispose individuals to frailty in old age. Consequently, the relaxation of natural selection in later life, combined with advances in medical care that prolong survival, contributes to an expanded phenotypic spectrum of frailty.

## 8 Reducing Acid Disturbance as Treatment of Frailty

Given that frailty is postulated to result from reduced acid‐buffering reserves, effective treatments must address both lowering the systemic acid load and enhancing the body's capacity for acid buffering and acid elimination.

Exercise is currently the best‐supported intervention to prevent and reverse frailty (Fried 2016; Mishra et al. 2024). Fried explained that exercise can improve the crosstalk among several interconnected physiological systems (Fried 2016). From the point of view of frailty driven by reduced acid buffers, we assume that aerobic exercise expands capillary networks and oxidative capacity in skeletal, cardiac, and respiratory muscles, thereby improving cardiopulmonary economy. This facilitates faster and more efficient removal of metabolic waste products and clearance of CO_2_ from the body (Green et al. 2017). Meanwhile, resistance exercise increases intramuscular buffering capacity, upregulates lactate/H^+^ transport, and improves ventilatory and renal regulation (Bishop et al. 2008). By inducing muscle hypertrophy (Wernbom et al. 2007) and enhancing mitochondrial respiration, resistance training improves skeletal muscle qualitatively and quantitatively (Porter et al. 2015). Because skeletal muscle is the main reservoir for non‐bicarbonate buffers and the primary site for metabolic acid production, these adaptations, collectively enhance the body's ability to maintain pH balance and delay fatigue. Empirically, multicomponent programs that combine moderate‐intensity endurance (e.g., brisk walking, cycling) with progressive resistance training and balance exercises yield the greatest gains in functional reserve, muscle strength, and resilience. Accordingly, the guideline of 2020 updated the recommendation from the 2010 guideline that all older people rather than only elderly with poor mobility should actively engage in multicomponent exercise programs at moderate or greater intensity at least 3 days a week (Bull et al. 2020). The beneficial effects of exercise still hold true for immobilized patients in that they can reduce immobilization‐induced osteoporosis and muscular atrophy.

Importantly, it is crucial to recognize that exercise itself acts as a metabolic stressor that can also induce metabolic acidosis, which in some cases may be severe and life‐threatening. The severity of exercise‐induced metabolic acidosis depends on the intensity and duration of activity as well as the individual's acid buffering capacity affected by factors such as bone mineral content, anemia status, muscle mass, cardiopulmonary, and renal function (Sue et al. 1988). Therefore, prescribing exercise as a treatment for frailty must be carefully individualized to ensure the metabolic cost does not overwhelm the patient's diminished acid buffer reserves.

On the other hand, protein supplementation has also been proposed as a treatment for frailty, but its standalone effects appear modest and inconsistent (Oktaviana et al. 2020) with more consistent benefits observed when combined with exercise (Yoshimura et al. 2025). This variability may be attributable, in part, to heterogeneity in amino acid composition. Sulfur‐containing amino acids (notably methionine and cysteine) are acid‐generating when metabolized and increase dietary acid load, whereas glutamate and aspartate—rich in legumes (soy, lentils, beans), nuts/seeds, and leafy vegetables—are more base‐producing (Patience 1990). Observational evidence from Japan indicates that dietary acid load is positively associated with frailty in elderly women (Kataya et al. 2018). Accordingly, depending on its amino acid composition, protein supplementation can be either acid‐contributing or acid‐reducing. Histidine, an essential amino acid, rich in meat, fish and some plant foods, is of particular interest: while neutral in terms of acid production, its imidazole side chains provide buffering capacity at physiological pH. Obese Chinese women with metabolic syndrome were found to have low serum histidine levels. In a 12‐week randomized, double‐blind controlled trial, daily supplementation of 4 g histidine reduced inflammation, oxidative stress and insulin resistance (Feng et al. 2013). These findings support a dual buffering/anti‐inflammatory mechanism of histidine supplementation and merit investigation as a targeted adjunct therapy for frailty, especially when combined with exercise. On the other hand, muscle carnosine content is known to decline with age, and in sarcopenia, restoring carnosine levels has been proposed as therapeutic target. Supplementation with carnosine (a histidine‐containing dipeptide) increases muscle carnosine contents by 60%–80% in young adults (Harris et al. 2006), directly improving performance during high‐intensity exercise (Hill et al. 2007). Similar beneficial effects were also observed in healthy elderly subjects, age 60–80 years (del Favero et al. 2012). In fact, carnosine has gained much research interest in geroscience in recent years because it was learned from smaller pilot studies and animal models to also have antioxidant, metal‐chelating, and anti‐glycation properties (Q. Wang et al. 2024). Furthermore, another double‐blinded placebo controlled randomized trial suggested that drinking alkalized water improved acid–base balance and anaerobic exercise performance in combat sport athletes (Chycki et al. 2018).

Given that frailty may fundamentally represent a systemic failure of acid‐buffering systems, narrow approaches such as hormonal supplementation or single‐target pharmacological interventions are unlikely to prevent or reverse the condition (Pazan et al. 2021). In the BiCARB (Witham et al. 2020) and VALOR‐CKD (Tangri et al. 2024) studies, recent large‐scale trials, even interventions aiming to target acid buffer by maintaining serum bicarbonate at 22–24 mmol/L levels failed to show improvements in muscle strength, physical function, or the rate of renal decline. These results suggest that frailty is a state of systemic buffer depletion; simply correcting extracellular bicarbonate concentrations does not necessarily resolve underlying systemic acid buffer deficits or repair the established cell damage in skeletal muscle or other tissues. Moreover, the use of sodium‐based buffers increased sodium load, which may induce complications such as volume expansion and hypertension, counteracting the potential metabolic benefits of acidosis correction.

Finally, medication optimization aimed at preserving renal acid–base handling may require careful consideration. As highlighted by Quaglia et al. (2014) clinicians face a therapeutic dilemma—while minimizing interference with the renin‐angiotensin‐aldosterone system (RAAS) preserves acid excretion capacity, RAAS inhibitors also provide essential cardiovascular and renal protection. This trade‐off is particularly crucial for older adults with diabetes mellitus, CKD, or congestive heart failure, as these comorbidities already predispose patients to type IV renal tubular acidosis and chronic acid retention (Quaglia et al. 2014). Consequently, management requires carefully balancing the benefits of target organ protection against the metabolic cost of systemic acid retention and thus acid buffering reduction.

## 9 Clinical Importance of Considering Individual Frailty Status

**FIGURE 1:**
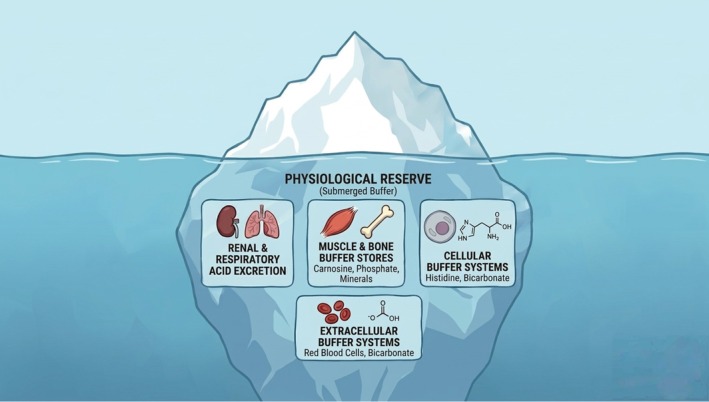
Acid buffering as the hidden physiological reserve underlying resilience. The iceberg represents the body's acid buffering reserve that determines apparent health. The submerged portion represents acid buffer in the cell (histidine‐rich proteins, phosphate, bicarbonate), in the plasma (hemoglobin, bicarbonate), bone matrix, and renal‐pulmonary systems for acid elimination. Together, these systems maintain acid–base balance under stress.

**FIGURE 2:**
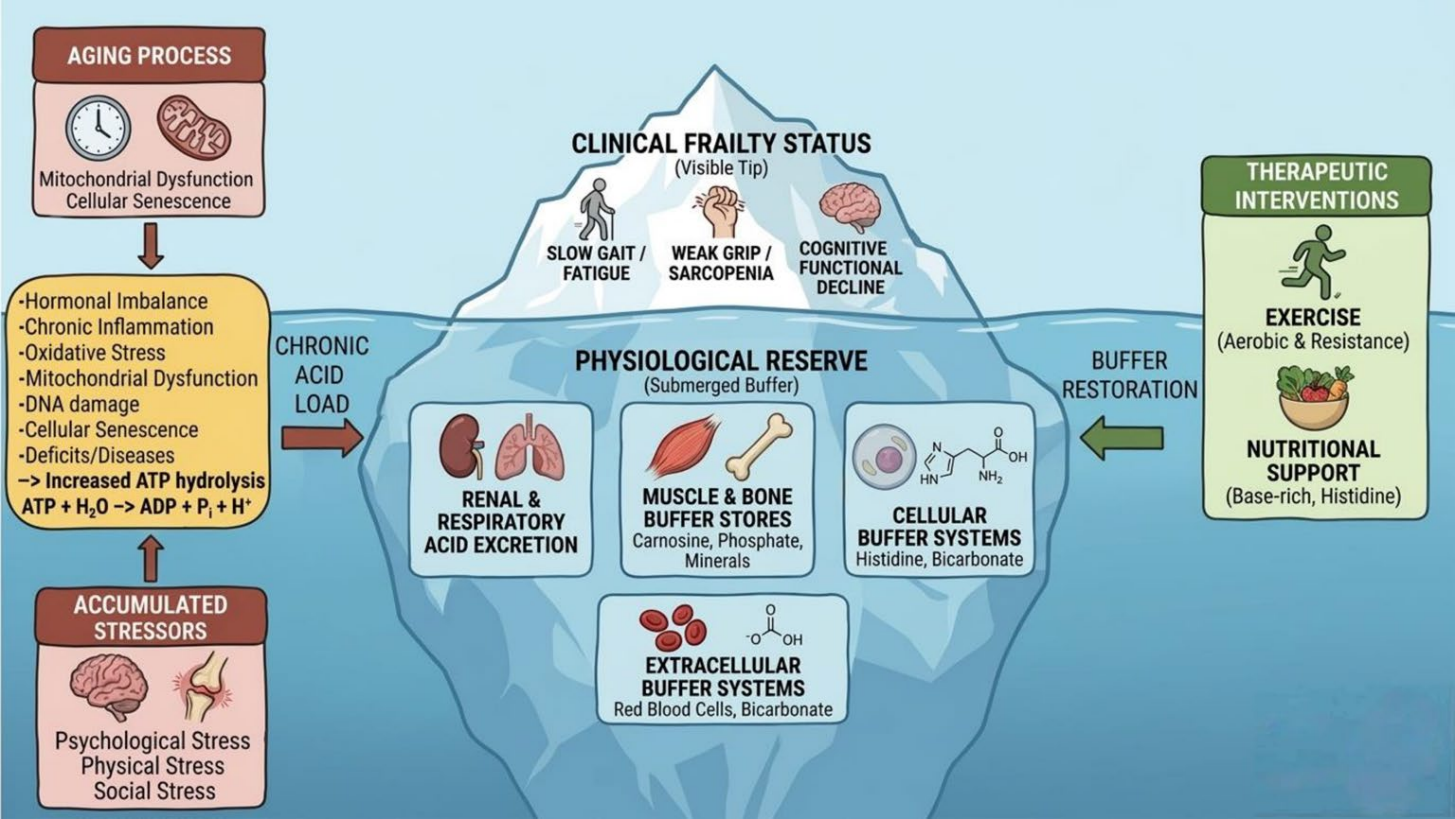
The Metabolic Buffer Model of Frailty. This figure illustrates frailty as a consequence of depleted physiological reserves, conceptualized as a “metabolic buffer” using an iceberg analogy. The left panel shows how the physiological aging process and accumulated stressors (psychological, physical, social) contribute to a chronic acid load, driven by factors like mitochondrial dysfunction, inflammation, and increased ATP hydrolysis. The central iceberg represents the body’s capacity to manage this load. The submerged part, the physiological reserve, consists of various acid buffer systems (renal and respiratory excretion, muscle and bone stores, cellular and extracellular buffers). When this buffering capacity is overwhelmed or depleted, the clinical frailty status emerges as the visible tip of the iceberg, manifesting in phenotypes such as slow gait, weak grip (sarcopenia), and cognitive/functional decline. The right panel outlines therapeutic interventions (exercise, nutritional support) aimed at buffer restoration. This metabolic model of frailty serves as a conceptual bridge connecting Rockwood’s deficit accumulation model of frailty from aging and accumulated stressors with Fried’s model of phenotypic frailty.

**FIGURE 3:**
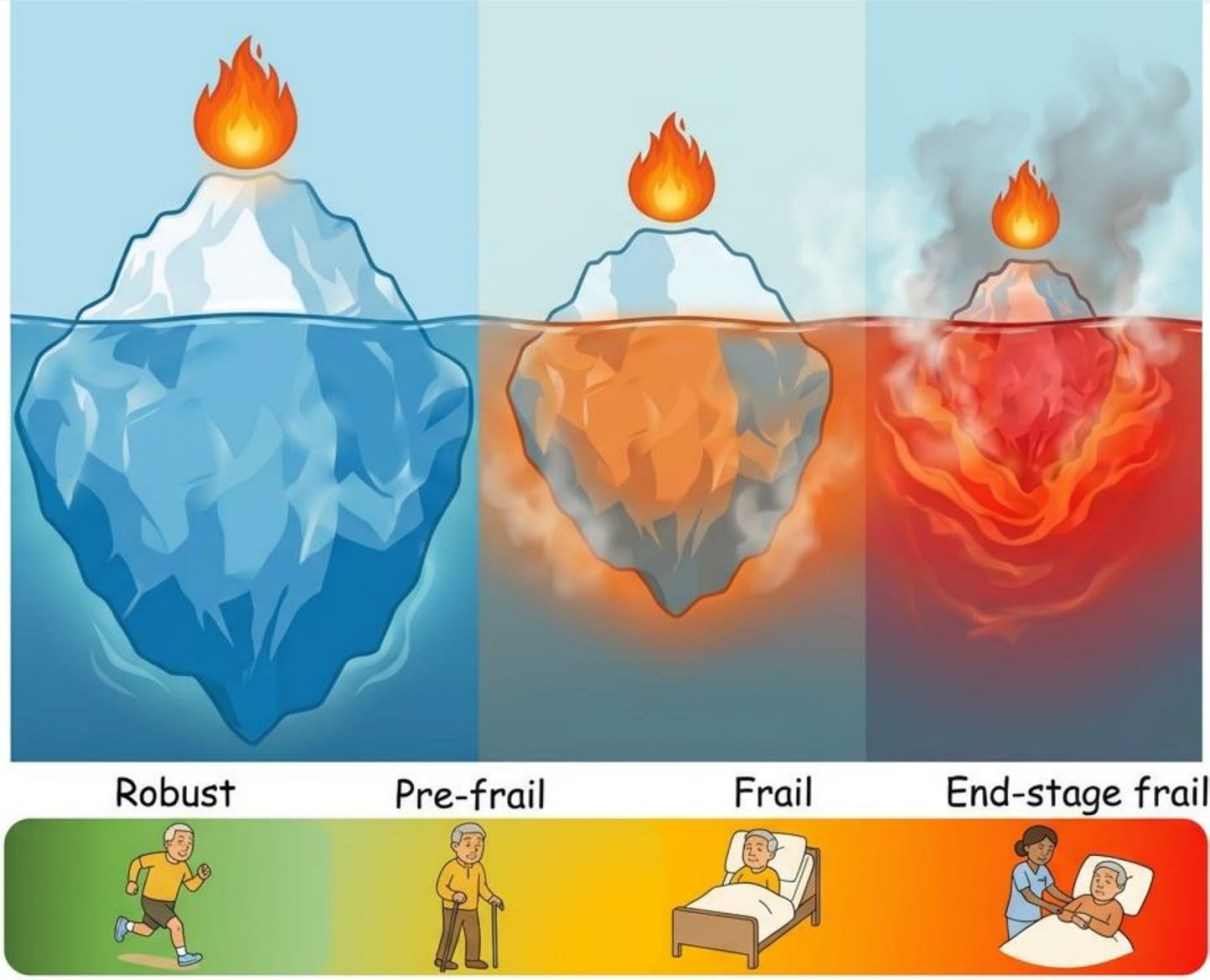
Shrinking iceberg as a continuum of frailty and energetic collapse. The progressively shrinking iceberg illustrates the decline of physiological and acid buffer reserves across the frailty spectrum‐ from robust to pre‐frail, frail to end‐stage frail. When acid buffer reserve is critically depleted as in end‐stage frailty, even a minor stressor can precipitate systemic instability, turmoil and metabolic collapse, represented by flames and turbulence around the iceberg. The visible tip represents apparent health—mobility, instrumental activities of daily living (IADL) and activities of daily living (ADL), cognition, and comorbidities—domains reflected in most clinical frailty scales.

Frailty‐related vulnerability to stress can thus be understood, in part, as a manifestation of impaired acid‐buffering capacity—symbolized by a melting iceberg. The submerged part of the iceberg symbolizes hidden physiological reserve‐ the body's acid buffering and adaptive systems that maintain homeostasis and enable repair during and after stressors (Figure 1). The visible tip corresponds to apparent status of health, roughly reflecting levels of mobility, instrumental activities of daily living (IADL) and activities of daily living (ADL), cognition, comorbidities, disease symptoms, domains on which most clinical frailty scales are based (K. T. Rockwood 2024). Chronic acid load and repeated stressors progressively erode the reserve, much like warming waters melting the iceberg from below (Figure 2). This process also triggers adaptive responses across amino acid metabolism, endocrine regulation and behavior, collectively aiming to stabilize pH balance. Clinically, this gradual erosion manifests as continuum from robustness to end‐stage frailty, reflected in varying degrees of instability when facing a similar stressor (Figure 3). With the reserve becoming severely diminished, even minor perturbations can precipitate systemic instability, symbolized as turmoil and collapse. Once dysregulation is initiated within the respiratory and renal systems that are essential for acid elimination, metabolic acidic byproducts rapidly accumulate, resulting in acidosis at cellular, tissue, and systemic levels. This internal disequilibrium accelerates the onset of diverse age‐related disorders, such as insulin resistance to diabetes, cancer, Alzheimer's disease (Decker et al. 2021), Parkinson's disease (Johannes Burtscher 2021), chronic kidney disease (Wesson et al. 2020), and osteoporosis, the incidences of which all increase with aging (Smit et al. 2024; Wilkerson 1947).

Clinical frailty status has been increasingly viewed as a sixth vital sign (Chung et al. 2025), indicating the individual's capacity to sustain physiological equilibrium in response to stress and illness. It reflects an overall physiological reserve to combat and to recover from stress. Adequate energy restoration during the recovery phase better prepares the body for future stressors. Unlike traditional vital signs such as temperature, pulse, respiratory rate and blood pressure—which reflect momentary physiological changes, frailty status represents a more dynamic and integrative measure of physiological resilience over time. This makes frailty assessment particularly valuable for prognostication when facing prolonged or intense challenges. For example, a frail individual is less likely to survive severe stress or illness compared to a non‐frail counterpart because of a reduced physiological reserve, impaired energy supply and diminished immune efficiency. Incorporating frailty status into clinical assessment enables a better prediction of the intensity of medical support required and provides insight into prognosis. Moreover, identifying frailty status can also guide the development of individualized interventions—such as tailored exercise or nutritional regimens, aiming at restoring reserve and resilience.

## 10 Conclusion

While frailty is widely recognized as a multifactorial syndrome involving inflammation, mitochondrial dysfunction, neuroendocrine dysregulation, we propose that these different contributors share a convergent metabolic downstream effect. Active inflammation, oxidative stress, and neuroendocrine dysregulation are bioenergetically expensive states, which increase ATP turnover and mitochondrial inefficiency, thereby generating sustained proton load. As acid–base homeostasis acts as a metabolic integrator of these upstream insults, we propose a model of metabolic buffer of frailty, which defines physiological reserve not only as the absence of disease or deficit but the total body acid buffering capacity to neutralize the protons imposed by stressors. When this buffering capacity is exhausted, the organism loses the pH stability to maintain normal functioning, precipitating the phenotypic decline observed in frailty.

Our model (graphical abstract) in a way unifies the two dominant, yet parallel lines of frailty perspectives: Rockwood's deficit accumulation model, which quantifies frailty as a summation of diverse health deficits, and Fried's Phenotypic model, which defines it as a clinical syndrome of energetic collapse. Different stressors identified in Rockwood's index‐ ranging from chronic organ disease to the persistent bioenergetic penalty of uncorrected sensory impairments like vision and hearing loss—share a convergent metabolic cost—they impose a continuous energy‐intensive demand on the organism. Furthermore, these deficits frequently trigger inflammatory and oxidative cascades, which amplify this energy consumption. Because ATP hydrolysis required to manage these deficits generates protons, the accumulation of deficits functionally translates into the accumulation of a chronic acid load.

In our model, the acid‐buffering capacity acts as the common biological currency that translates accumulated deficits into phenotypic failure. Chronic acid load progressively exhausts the body's non‐bicarbonate buffer. As these fundamental reserves become depleted and serum bicarbonate trends downward, the system reaches the tipping point of Fried's energetic collapse. In this state, sarcopenia is not only a feature of aging, but an active catabolic compensation, which degrades muscle to generate ammonia to maintain acid–base homeostasis. Thus, our model provides the mechanistic unification: Rockwood's deficits represent sources of metabolic stress, reduced acid buffering capacity represents depletion of reserves, and Fried's phenotype represents the final functional failure, manifested as a clinical syndrome.

## 11 Limitations and Future Direction

A current challenge in validating the role of acidosis in frailty is the lack of longitudinal data tracking pH evolution against frailty onset, making it difficult to determine whether acidosis is a driver of frailty or a marker of severity of frailty. Furthermore, current clinical assessment of acid stress is often limited to serum bicarbonate or net renal acid excretion. These static markers, however, may not faithfully reflect intracellular pH dynamics or the status of intracellular or tissue buffers, which vary significantly among individual cells and tissues. Because serum pH is aggressively guarded at the expense of intracellular reserve, serum bicarbonate may remain normal even when intracellular and tissue buffers have already been much depleted, a state of “compensated structural failure”.

To test our metabolic model of frailty, on the other hand, requires the quantification of the total acid buffer capacity. It should be viewed as a composite index, integrating structural reserves, mainly muscle, bone and RBCs with the dynamic clearance capacity by the renal and pulmonary systems. Frailty emerges when these capacities fall below a critical threshold. In this state, the body maintains homeostasis by sacrificing its own structural integrity, creating a feedback loop of muscle wasting and skeletal decline. This exhausted reserve renders the individuals prone to systemic collapse even when triggered by minor stressors.

## Author Contributions

Wan‐Hui Liao conceived and drafted the perspective and the creation of the graphical abstract and supporting figures. All authors contributed to conceptual development, critical revision, and final approval of the perspective.

## Funding

The authors have nothing to report.

## Conflicts of Interest

The authors declare no conflicts of interest.

## Data Availability

The authors have nothing to report.
